# Private Psychiatric Care in Bangladesh: Clinical Complexity, Risk, and Service Pathways

**DOI:** 10.1192/bjo.2026.11599

**Published:** 2026-06-30

**Authors:** Mehtab Ghazi Rahman, Nuzhat Tabassum Urbi, Lisanul Hasan, Rannia Shehrish, Moostafi Tarannum Jhilik

**Affiliations:** 1 Central & North West London NHS Foundation Trust, London, United Kingdom; 2 Neuroscience & Psychiatry Hub Ltd., Dhaka, Bangladesh; 3 Neuroscience & Psychiatry Hub Ltd., Dhaka, United Kingdom

## Abstract

**Aims:**

Bangladesh is one of the most densely populated countries globally and carries a substantial burden of mental illness. National survey data estimate the prevalence of mental disorders at approximately 16.8–18.7% among adults and 13–14% among children, alongside a marked treatment gap, with over 90% of affected individuals not receiving formal mental healthcare. Public psychiatric services remain limited in capacity, are geographically concentrated in urban centres, and are often characterised by delayed presentation, prolonged inpatient stays, and poor continuity of care. In this context, private psychiatric hospitals are increasingly accessed in urban areas. There is little published evidence describing the service role, clinical acuity, and patient profile of private-sector psychiatry in low- and middle-income countries.

Aim: To describe the clinical, sociodemographic, and service characteristics of patients admitted to a private psychiatric hospital in Bangladesh and to contextualise these findings within international psychiatric service models.

**Methods:**

A retrospective descriptive review was conducted of consecutive inpatient admissions to The HUB over a ten-month period (April 2025 – January 2026). Patients accessing only outpatient or triage services were excluded. Anonymised data were extracted from routine clinical assessment documentation, including age, gender, socioeconomic background, education and employment status, psychiatric diagnosis, prior treatment history, risk factors, legal status, and admission pathways. Data were analysed descriptively.

**Results:**

The cohort comprised 44 consecutive inpatient admissions. Patients were predominantly adults, with a male predominance of approximately 60%. Most patients were from upper or upper-middle socioeconomic backgrounds and demonstrated relatively high educational attainment, including undergraduate and postgraduate qualifications. Employment status was heterogeneous, encompassing professionals, business owners, students, and unemployed individuals.

Majority admissions reflected established psychiatric illness rather than first-episode presentations. The majority of patients had a documented history of prior psychiatric treatment, frequently within private healthcare settings. Notably, over three-quarters had previously received psychiatric care outside Bangladesh, indicating prominent international care pathways. Diagnostic profiles included mood disorders, psychotic disorders, anxiety-related disorders, and substance-related conditions, consistent with a broad general adult psychiatry case mix.

Clinically significant risk was common. Approximately one in six patients had a history of self-harm, and a similar proportion presented with behavioural disturbance or aggression. Suicidal ideation was documented in a subset of cases, although recording was variable across assessments. Admissions occurred via both planned and acute or crisis pathways. While many patients were admitted voluntarily, some required higher levels of containment and intensive risk management.

**Conclusion:**

This study demonstrates that private psychiatric hospitals in Bangladesh manage patients with substantial clinical complexity, risk, and prior treatment exposure, challenging assumptions that private-sector psychiatry predominantly addresses low-acuity or elective care. The observed service and clinical profile shows important parallels with private services in high-income countries. These findings support the inclusion of private-sector data in global mental health research and highlight private hospitals as important components of mental health systems where public capacity is limited.

